# Bioinspired Scaffold Action Under the Extreme Physiological Conditions of Simulated Space Flights: Osteogenesis Enhancing Under Microgravity

**DOI:** 10.3389/fbioe.2020.00722

**Published:** 2020-07-08

**Authors:** Elisabetta Avitabile, Laura Fusco, Silvia Minardi, Marco Orecchioni, Barbara Zavan, Acelya Yilmazer, Martina Rauner, Proto Pippia, Ennio Tasciotti, Lucia Gemma Delogu

**Affiliations:** ^1^Department of Chemistry and Pharmacy, University of Sassari, Sassari, Italy; ^2^Department of Chemical and Pharmaceutical Sciences, University of Trieste, Trieste, Italy; ^3^Fondazione Istituto di Ricerca pediatrica Cittá della Speranza, Padua, Italy; ^4^Cancer Research Department, Sidra Medicine, Doha, Qatar; ^5^Center for Musculoskeletal Regeneration, Houston Methodist Research Institute, Houston, TX, United States; ^6^Department of Medical Sciences, University of Ferrara, Ferrara, Italy; ^7^Maria Cecilia Hospital, GVM Care & Research, Ravenna, Italy; ^8^Department of Biomedical Engineering, Ankara University, Ankara, Turkey; ^9^Stem Cell Institute, Ankara University, Ankara, Turkey; ^10^Department of Medicine III, Center for Healthy Aging, Technische Universität Dresden, Dresden, Germany; ^11^Department of Physiological, Biochemical and Cellular Science, University of Sassari, Sassari, Italy; ^12^Department of Nanomedicine, Houston Methodist Research Institute, Houston, TX, United States; ^13^Department of Biomedical Science, University of Padua, Padua, Italy

**Keywords:** stem cells, nanomaterials, scaffolds, microgravity, random positioning machine, bone, tissue regeneration, space biology

## Abstract

Prolonged exposure to microgravity (MG) during long-duration space flights is known to induce severe dysregulation of osteoblast functions connected to a significant bone loss, similar to the condition induced by osteoporosis. Hence, we here present MG as a promising model to challenge the effectiveness of new scaffolds designed for bone regeneration in counteracting bone loss. To this end, we carried out an integrative study aimed to evaluate, in the extreme condition of Random Positioning Machine-simulated MG, the osteoinductive potential of nanocrystalline magnesium-doped hydroxyapatite/type I collagen composite scaffold (MHA/Coll), that we previously demonstrated to be an excellent tool for bone tissue engineering. Initially, to test the osteoinductive properties of our bioinspired-scaffold, MHA/Coll structure was fully characterized under MG condition and compared to its static counterpart. Human bone marrow-derived mesenchymal stem cells were used to investigate the scaffold biocompatibility and ability to promote osteogenic differentiation after long-duration exposure to MG (up to 21 days). The results demonstrate that the nanostructure of MHA/Coll scaffold can alleviate MG-induced osteoblast dysfunction, promoting cell differentiation along the osteogenic lineage, with a consequent reduction in the expression of the surface markers CD29, CD44, and CD90. Moreover, these findings were corroborated by the ability of MHA/Coll to induce the expression of genes linked to osteogenesis, including alkaline phosphatase and osteocalcin. This study confirmed MHA/Coll capabilities in promoting osteogenesis even in extreme long-term condition of MG, suggesting MG as an effective challenging model to apply in future studies to validate the ability of advanced scaffolds to counteract bone loss, facilitating their application in translational Regenerative Medicine and Tissue Engineering.

## Graphical Abstract

Schematic representation of MHA/Coll function in microgravity condition to counteract bone loss dysfunction.

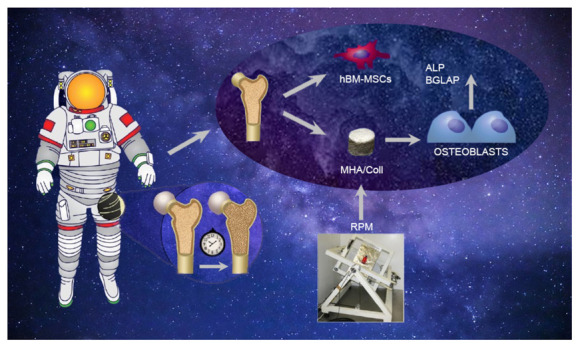

## Introduction

One of the major health problems that human face during long-duration space flights is accelerated aging and, as a consequence, a significant bone loss, quantified as total bone mineral density (BMD), that may ultimately affect the quality of life (Manzey and Lorenz, 1999; LeBlanc et al., 2000). Progressive bone loss under microgravity (MG) conditions is related to an impairment of osteoblast and an increase of bone resorption with a significant decrease in osteogenic gene expressions that are ordinarily connected to a normal bone resorption. Several experiments of Russian space station Mir, American and European missions, conducted by astronauts or simulation models, have described the variation of both pre- and post-flight bone loss (Durnova et al., 1996; Heer, 2002; Lang et al., 2017; Maude et al., 2017). During the missions on the International Space Station (ISS) it was found that such loss is usually in the range between 3 and 10% (Kozlovskaya and Grigoriev, 2004; Nagaraja and Risin, 2013). Continuous bone loss of astronauts during or after space missions can increase the risk of developing fractures in the skeletal framework. Different studies have described the negative effect of MG that acts by altering the bone structure of *ex vivo* models and *in vivo* in mice (Kaplansky et al., 1991; Vanloon et al., 1995; Maroothynaden and Hench, 2006; Zhang et al., 2018), demonstrating that simulated MG is the main contributor to bone loss when compared to other extreme physiological conditions associated with space travels, such as radiation and ultradian rhythms (Zhang et al., 2018). Indeed, it is well-known that MG can induce bone loss in terms of BMD decrease of about 2% in 30 days, an effect comparable to that induced by postmenopausal osteoporosis in women in about a year (Riggs et al., 1998). It has been demonstrated that the first adverse effects observed during short-duration space missions are loss of calcium and bone changes, occurring within 10 and 20 days after beginning the space flight, respectively (Rambaut and Johnston, 1979; McCarthy et al., 2000).

Since many of the experiments carried out so far were aimed at evaluating the effect of short-duration MG simulation on cells (Peana et al., 2004; Degan et al., 2005; Martinelli et al., 2009; Tauber et al., 2015), several studies are now exploring the impact of MG on cells and physiology under long-duration MG condition (Kornilova et al., 2006; Koryak, 2014; Chen et al., 2017; Harris et al., 2017; Blaber and Parker, 2018). Recently, Tavella et al. (2012) investigated the alterations of bone microarchitecture using animal models during 91 days on the ISS. The authors described that MG can induce bone loss due to a decrease in bone deposition and an increase of bone resorption in wild type and pleiotrophin-transgenic mice. It has been demonstrated that short- and long-duration spaceflights cause the dysregulation of stem cells functions which leads to the inability of cells to repair and regenerate lesions (Blaber E. et al., 2014, 2015). These findings indicate that MG induces several modifications in osteoblasts and osteoclasts in terms of cell morphology, proliferation, and differentiation (Dai et al., 2007, 2013; Xu et al., 2017).

Experiments carried out on osteoblastic cell cultures by using a tridimensional clinostat as Random Positioning Machine (RPM) to simulate MG, are focused on the investigation of spaceflight-related osteoblastic dysregulation (Pardo et al., 2005; Patel et al., 2007). Recently, an innovative study by Shi et al. (2017) reported that primary cilia (key sensor and functioning organelles) of rat calvaria osteoblasts vanished after MG exposure. To address the problem of the modification of osteoblasts and osteoclasts in MG, several groups have used RPM or Rotary wall vessel bioreactors (RWV) to simulate MG conditions (Nakamura et al., 2003; Ontiveros and McCabe, 2003), demonstrating the gene expression dysregulation of important osteogenic-related osteoblastic genes, such as alkaline phosphatase (ALP) and osteocalcin (BGLAP) (Pardo et al., 2005; Hu et al., 2015; Grimm et al., 2018).

Thanks to the advent of nanotechnology, new nano-structured biomaterials and scaffolds have been produced for bone regeneration (Sechi et al., 2014; Minardi et al., 2015, 2019). In this context, we have recently developed a bioinspired three-dimensional (3D) nanocrystalline magnesium-doped hydroxyapatite/type I collagen composite scaffold (MHA/Coll) (Minardi et al., 2015), demonstrating that its composition and nanostructure closely recapitulated that of human trabecular bone. *In vitro*, MHA/Coll triggered the osteogenic differentiation of primary human bone marrow mesenchymal stem cells (hBM-MSCs), inducing the early expression of crucial osteogenesis-associated marker genes, such as ALP and BGLAP. Moreover, due to the high degree of biomimicry of MHA/Coll, enhanced bone augmentation and regeneration was achieved *in vivo*, in both an ectopic (Minardi et al., 2015), and orthotopic model in large animal models (rabbit) (Minardi et al., 2019).

Previous research of our and other groups demonstrated the key role of nanotechnologies and regenerative medical approaches via the use of nanomaterials and biomaterials to compensate some of the dysregulations caused by MG conditions (Grattoni et al., 2012; Crescio et al., 2014). Therefore, we here challenged the biomimicry and bone regenerative properties of MHA/Coll in the extreme condition of MG, evaluating its ability to counteract spaceflight osteoblast dysregulation with the aim of proposing MG as a model to validate the effectiveness of new scaffolds designed for bone regeneration in promoting new bone formation, due to the negative effects induced by MG on bone functions which are similar to those elicited by medical conditions (e.g., osteoporosis). To this end, the architecture and the osteoinductive potential of our 3D bioinspired scaffold were investigated using RPM to simulate MG, evaluating its morphology and ability to induce hBM-MSCs differentiation into osteoblast under long-time exposure to MG condition. Cell morphology, viability, and differentiation of hBM-MSCs cultured on MHA/Coll were determined. Furthermore, the expression of a wide variety of osteoblastogenesis-associated genes was analyzed in comparison to uninduced controls under MG and on hearth conditions. Our goal is to set the ground for new solutions based on MG conditions to evaluate the ability of advanced scaffolds to counteract bone loss, as an extreme physiological condition inducing effects similar to those occurring in medical conditions (e.g., osteoporosis), facilitating their application in translational Regenerative Medicine and Tissue Engineering.

## Materials and Methods

### Scaffold Fabrication and Characterization

MHA/Coll was fabricated as described elsewhere (Minardi et al., 2015). Briefly, an acidic solution of bovine type I collagen (Nitta Casing Inc.) was prepared at a concentration of 10 mg/ml in acetate buffer at pH 3.5. An aqueous solution of H_3_PO_4_ (40 mM) was added to 40 g of the acetic collagen gel, and dropped in a solution of Ca(OH)_2_ (40 mM) and MgCl_2_⋅6H_2_O (2 mM) in DI water. The material was crosslinked in an aqueous solution of 1,4-butanediol diglycidyl ether (BDDGE) (2.5 mM). The resulting slurry was molded in 48-well plates at a thickness of 3 mm. Finally, the slurry was lyophilized through an optimized protocol, to generate the desired porosity and pore size. Non-mineralized collagen scaffolds (Coll) were also synthesized and used as controls (Minardi et al., 2014, 2015). All chemicals were purchased from Sigma Aldrich. The monolithic scaffold was imaged by scanning electron microscopy (FEI Quanta 400 SEM). The scaffolds were sputter-coated with 10 nm of Pt/Pd, via a Plasma Sciences CrC-150 Sputtering System (Torr International, Inc), and imaged at a voltage of 7.5 kV. Fourier-transformed Infrared spectroscopy (FTIR) was performed through a Nicolet 4700 Spectrometer. Sixty-four runs were performed per sample (*n* = 3). Spectra were analyzed by the software EZ OMNIC (Nicolet). The amount and thermal properties of mineral phase nucleated on the organic template (type I collagen) was assessed by thermal gravimetric analysis – differential scanning calorimetric (TGA-DSC). The samples (*n* = 3) were placed in alumina pans and subjected to a heating ramp from 25 to 800°C, at 10°C/min. A Q-600 TGA was used (TA Instruments).

### hBM-MSC Culture

Bone marrow aspirates were collected from healthy donors of both genders (22–49 years old) following Institutional Review Board approval (Uniklinikum, Dresden, Germany). Written informed consent was obtained from all the donors. Bone marrow aspirate was diluted 1:5 in PBS. A 20 ml aliquot was layered over a biocoll solution (1.077 g/ml, Biochrom) and centrifuged at 550 g for 30 min at room temperature to separate mononuclear cells from anuclear red blood cells (RBCs). Following centrifugation, RBCs were at bottom of the tube and mononuclear cells, including the desired hBM-MSCs, were collected at the interface above the band of biocoll. To isolate hBM-MSCs, their adherent properties were exploited by culturing the mononuclear cells in 75 cm^2^ flasks in Dulbecco’s modified Eagle medium (DMEM)-low glucose supplemented with 1% of penicillin/streptomycin solution and 10% fetal calf serum at 37°C under a humidified 5% CO_2_ atmosphere. After 24 h, cells were washed with phosphate buffered saline (PBS) to remove non-adherent cells, such as hematopoietic cells, representing a relatively large portion of the bone marrow. Subsequently, the medium was changed every 2 days, and after 2 weeks the cultures were 90% confluent. To induce osteogenic cell differentiation, hBM-MSCs were cultured in inducing medium (StemPro^®^ Osteogenesis Differentiation Kit, Gibco) supplemented with 25 mM HEPES buffer solution [4-(2-hydroxyethyl)-1-piperazineethanesulfonic acid, Gibco]. During the experiments, the medium was changed every 2 days.

### Microgravity Simulation

MG was simulated by a random position machine (RPM, Fokker, Netherlands). The RPM is a 3D clinostat able to produce a multilateral gravitational simulation when the samples are set in the center of the machine. A computerized program was used to create random movements and slow rotation of the two axes of the RPM to provide MG simulation (0 × *g*). Static cell cultures were placed in the basement of the RPM to simulate gravity (G) condition (1 × *g*).

### MHA/Coll Structural Characterization and Cell Morphology Under Microgravity Condition

The effects of long-duration MG exposure (21 days) on MHA/Coll structure and hBM-MSC morphology were evaluated using SEM. For cell morphology analysis, undifferentiated hBM-MSCs were harvested, and a 30 μl drop containing 35 × 10^4^ cells was seeded in the center of MHA/Coll scaffolds and kept in the incubator for 10 min. Culture medium was then added to each well and the effects induced by long-term exposure to MG were evaluated after 21 days. For SEM morphologic investigation, the upper surface of the scaffold was analyzed after 21 days. The samples were fixed in 2.5% glutaraldehyde in 0.1 M phosphate buffer (pH 7.2) and post-?xed in 1% Osmium Tetroxide (OsO4), dehydrated in a graded acetone series and dried by critical point method in an Polaron Jumbo apparatus (Polaron Equipment, Watford, United Kingdom) coated with gold in an Edwards S150A Sputter Coater unit (Edwards, Crawley, United Kingdom). The specimens were examined with a Zeiss DSM 962 SEM (Zeiss, Oberkochen, Germany).

### hBM-MSCs Viability Assay

Biocompatibility was evaluated via cytotoxicity assay in hBM-MSCs on MHA/Coll scaffold by the 7-amino-actinomycin D (7-AAD) staining (BD Bioscience, San Josè, CA, United States). When excited by 488 nm laser light, 7-AAD fluorescence is detected in the far-red range of the spectrum (650 nm long-pass filter). Late apoptotic and necrotic cells with compromised membranes allow the passage of this dye into the nucleus. hBM-MSCs were seeded into scaffolds and cultured for 7, 14, and 21 days under G or MG conditions. To collect cells, the scaffold was then washed three times with PBS and digested using 2 mg/ml collagenase I (Life Technologies) diluted in cell media without FBS (1-h incubation at 37°C). Subsequently, cells were washed with PBS to eliminate collagenase and stained with 7-AAD for 20 min in the dark. Finally, cells were suspended in PBS 1× solution and analyzed by Flow cytometry (FACS Canto II, BD Biosciences, Mountain View, CA, United States). At least three samples per group were used.

### hBM-MSCs Osteogenic Differentiation Under Microgravity Condition

Undifferentiated hBM-MSCs were harvested and a 30 μl drop containing 35 × 10^4^ cells was seeded in the center of MHA/Coll scaffolds and kept in incubator for 10 min. Inducing medium was then added to each well and the osteogenic potential of MHA/Coll was evaluated after 7, 14, and 21 days. under G or MG conditions. hBM-MSCs cultured in 2D conditions, either exposed to inducing media (induced-MSC) or kept in standard media (ctrl-MSC) were used as a positive and negative control, respectively. RPM-cultures were mounted horizontally in the center of the RPM at 37°C. As a control grown under G condition (1 × *g*), the same number of samples was placed in the same room of the RPM at 37°C in horizontal position. The results are expressed as % of positive cells and are the mean of three independent experiments.

### Gene Expression Analysis

Total RNA was isolated from cells using TriZol Reagent (TriZol, Invitrogen, Carlsbad, CA, United States). RNA purity and concentration were measured with Nanodrop Spectrometer (NanoDrop^®^ ND1000).

cDNA synthesis was performed using Superscript IV Reverse Trascriptase kit following the manufacturer protocol (Life Technologies). Amplification was performed using TaqMan probes and TaqMan^®^ Fast Advanced Master Mix (Applied Biosystems) to evaluate the expression of the osteogenic specific genes alkaline phosphatase (ALP, Hs01029144_m1) and osteocalcin (BGLAP; Hs01587814_g1). The gene expression analysis was performed comparing hBM-MSCs cultured on MHA/Coll and hBM-MSCs cultured in osteogenic media (induced-MSC), or uninduced (ctrl-MSC), under G and MG conditions. Glyceraldehyde 3-phosphate dehydrogenase (GAPDH; Hs02758991_g1) was used to normalize gene expression data respect to ctrl-MSC. To identify the expression of 84 osteogenesis genes, RT2 Profiler Polymerase Chain Reaction (PCR) Array (PAHS-026ZD, Superarray Bioscience Corporation, Frederick, MD) was applied. Amplifications on plates were set using a CFX96 Real Time instrument (Bio-Rad). Results are the mean of three independent experiments.

### Statistical Analysis

Data analyses were performed using Prism GraphPad software. Statistics for experiments were performed using a One-Way ANOVA. In all cases, ^∗^ was used for *p* < 0.05, ^∗∗^ for *p* < 0.01, ^∗∗∗^ for *p* < 0.001, and ^****^ for *p* < 0.0001. Values were expressed as mean ± SD. Flow cytometry data were analyzed with FACS Diva software (BD-Bioscience Mountain View, CA, United States). Osteo-gene array data were calculated by the comparative threshold cycle method. Data analysis was performed by RT2 profiler PCR array data analysis software^1^. All experiments were performed at least in triplicate.

## Results and Discussion

### Characterization of MHA/Coll

**FIGURE 1:**
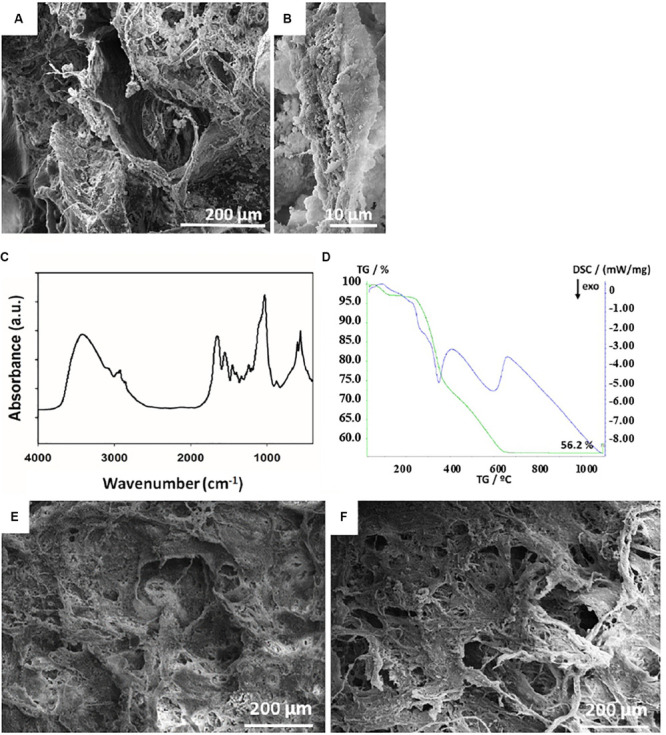
Scaffold architectures. SEM structural characterization of MHA/Coll scaffold **(A)**. The higher magnification micrograph of MHA/Coll **(B)** shows the collagen fibers with a full mineralization and an amorphous apatite phase. FTIR spectra showing the chemical interaction between the mineral phase and the type I collagen fibers of MHA/Coll **(C)**. Evaluation of the mineral phase on MHA/Coll by TGA-DSC analysis **(D)**. SEM micrographs of MHA/Coll structure evaluated after 21 days under gravity (G, 100×) **(E)** or microgravity (MG, 100×) conditions **(F)**.

The surface architecture and structure of Coll and MHA/Coll were evaluated by SEM (Figure 1). The lower magnification images revealed the porous anisotropic nature of MHA/Coll (Figure 1A) and their full mineralization compared to a Coll scaffold (see Supplementary Figure 1). The nano-MHA phase nucleated on the collagen fibers clearly did not appear crystalline, but rather amorphous, at higher magnification, as previously accomplished through the same bioinspired synthesis process (Minardi et al., 2015; Figure 1B). The chemical interaction between the mineral phase and the type I collagen fibers of MHA/Coll was further confirmed by FTIR spectroscopy (Figure 1C), where a shift from 1340 to 1337 cm^–1^ in the band corresponding to the stretching of carboxylate (COO^–^) group of collagen was observed, as expected. The TGA-DSC analysis showed that the overall mineral phase content in MHA/Coll was approximately 56 wt%, which is comparable to that of natural trabecular bone (Minardi et al., 2015; Figure 1D).

Finally, MHA/Coll structure was evaluated by SEM after 21 days under G (Figure 1E) and MG conditions (Figure 1F).

### Scaffold Structure and Cell Morphology Under Microgravity Condition

**FIGURE 2:**
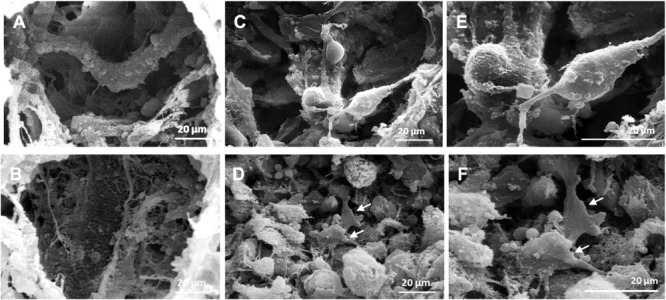
SEM micrographs of MHA/Coll structure and cells morphology. SEM micrographs of MHA/Coll architecture under G condition **(A)** and MHA/Coll after MG exposure **(B)** (1000×). Induced hBM-MSCs morphology in the center of the scaffold surface after 21 days under G condition **(C)** and simulated MG condition **(D)**. Induced hBM-MSCs connected together and attached onto the fibers of the scaffold under G condition **(E)** and after MG exposure **(F)** (2000×). White arrows indicate changes in cell morphology induced by MG exposure.

We previously found that MHA/Coll was able to mimic the osteogenic niche of human trabecular bone having osteogenic and osteoinductive properties (Minardi et al., 2015). MHA/Coll is synthesized through a sophisticated bioinspired nanotechnological process, which recapitulates the chemical, physical, morphological and structural control mechanisms of the natural biomineralization process (Mann, 2001). During the synthesis, a partial substitution of calcium with magnesium ions in the apatite lattice allows an amorphous nanostructured apatite, which closely mimics the early osteogenic niche (De Bruijn et al., 1992). On the basis of our previous findings, the effects induced by long-term exposure to MG (21 days) on MHA/Coll structure and hBM-MSCs morphology were evaluated by SEM (Figure 2). Under G condition MHA/Coll showed a fibrous structure (Figure 2A). On the contrary, MHA/Coll fibers under long-term MG condition were remodeled, resulting in slight compression and collapsing of the pores (Figure 2B), compared to the untreated scaffolds (Figures 1A,B). Figure 2C represents induced-hBM-MSC morphology in the center of the scaffold surface under G condition, while morphology changes induced by long exposure to simulated MG are shown in Figure 2D. Higher magnification revealed induced-hBM-MSCs attached to the mineralized nanostructured fibers of the scaffolds under G condition (Figure 2E). Under MG conditions, hBM-MSCs’ morphology appeared different compared to the untreated controls (Figure 2F). In particular, cells seemed to have partially lost their typical spindle-like shape presenting a flattened form (white arrows, Figures 2D,F). This effect is in line with previous studied carried out on stem cells in MG morphology (Zhu et al., 2014; Zhang et al., 2015; Xue et al., 2017).

### Cell Viability on the Bio-Scaffold Under Microgravity

The effect of simulated MG on the MHA/Coll biocompatibility was evaluated on hBM-MSCs. To this end, cells were cultured for 7, 14, and 21 days on the scaffold under G or MG conditions and cell viability was analyzed following 7-AAD staining, a fluorescent chemical compound with strong affinity for DNA, by flow cytometry.

**FIGURE 3:**
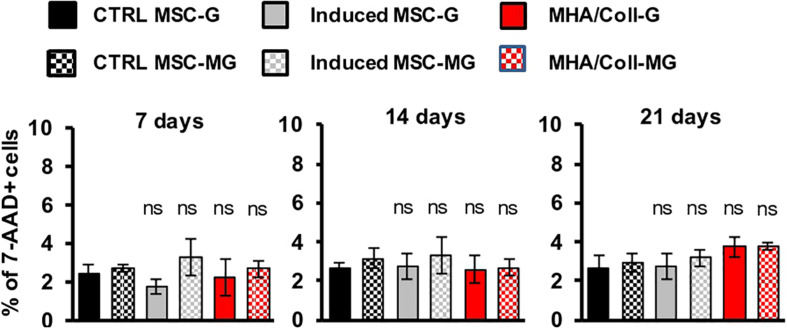
Viability assay of hBM-MSCs cultured on MHA/Coll scaffolds. hBM-MSCs were cultured on MHA/Coll scaffolds (red) for 7, 14, and 21 days under G or MG conditions. Cell viability was evaluated by flow cytometry after staining with 7-AAD. The results are expressed as % of positive cells (7AAD+) compared to uninduced-MSC (ctrl-MSC, black) and are reported as mean ± SD, *N* = 3.

Overall, the obtained results showed no significant effect on cell viability neither under G nor MG conditions, between MHA/Coll in comparison to the controls after 7, 14, and 21 days; therefore, demonstrating the high biocompatibility of our MHA/Coll scaffold even in the extreme condition of MG (Figure 3).

### Mesenchymal Stem Cell Differentiation Markers Under Microgravity Condition

The potential of hBM-MSCs to differentiate along osteogenic lineage (De Ugarte et al., 2003) and the consequent reduction in the expression of most surface markers related to MSCs differentiation is well-known (Halfon et al., 2011). Therefore, the influence of long-term MG simulation on hBM-MSCs differentiation in induced 3D and 2D cultures compared to the uninduced controls was evaluated by the expression of specific cell surface differentiation markers. To this end, hBM-MSCs seeded on MHA/Coll scaffold, induced-MSC and ctrl-MSC were cultured for 7, 14, and 21 days under G or MG conditions and the expression of hBM-MSCs surface markers CD29 (a β1 integrin associated with late antigen receptors) and CD44 (a hyaluronic acid/fibronectin receptor involved in hematopoietic stem cell adhesion, mobilization and proliferation), was evaluated by flow cytometry.

**FIGURE 4:**
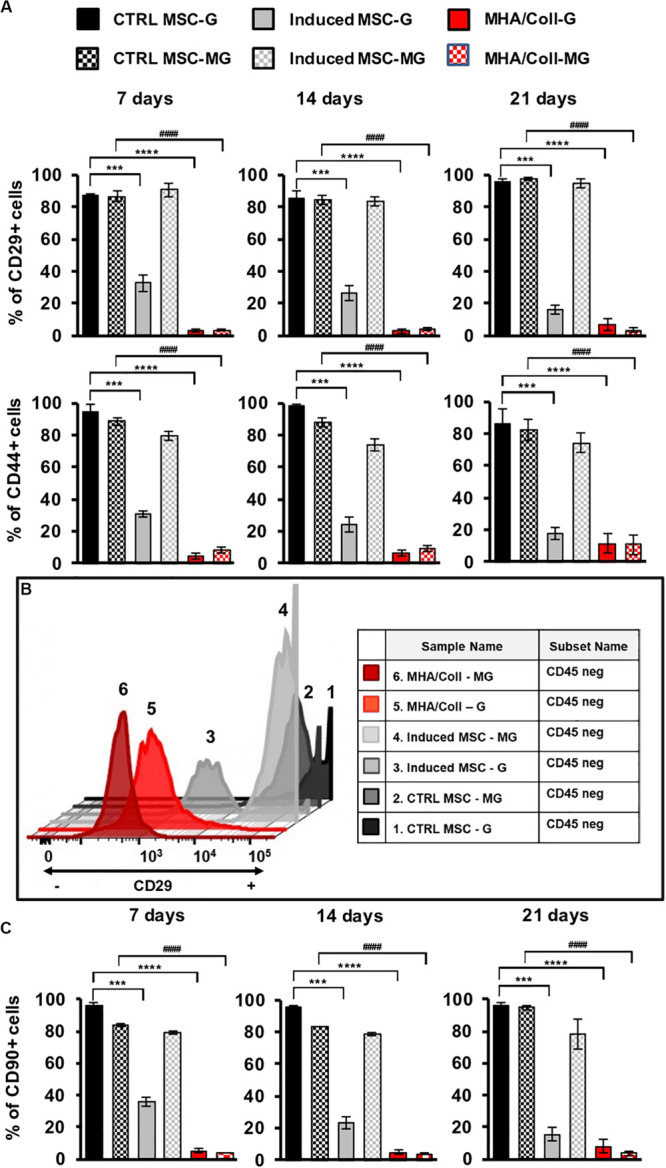
MHA/Coll-induced hBM-MSCs differentiation under MG condition. hBM-MSCs osteogenic differentiation was investigated in MHA/Coll scaffolds after 7, 14, and 21 days under G or MG conditions. The levels of the differentiation markers CD29 and CD44 were evaluated by flow cytometry. Uninduced-MSC (black) and induced-MSC (gray) were used as negative and positive control, respectively, for induced hBM-MSCs cultured on scaffolds **(A)**. Differentiation plot displaying hBM-MSCs, which are negative for CD45 marker, and differentiated cells, which showed the loss of the differentiation marker CD29. Uninduced-MSC (black) and induced-MSC (gray) were used as negative and positive control, respectively, for induced hBM-MSCs cultured on scaffolds **(B)**. Cell differentiation of hBM-MSCs cultured on MHA/Coll scaffolds was investigated after 21 days evaluating the expression of CD90 as a differentiation marker toward osteogenic lineage **(C)**. The results are expressed as % of positive cells and are the average of three independent experiments. Significant differences: ***p < 0.001 and ****p < 0.0001, vs. ctrl-MSC under G condition; ^####^*p* < 0.0001, vs. ctrl-MSC under MG condition (Two-way ANOVA).

Intriguingly, a significant reduction (*p* < 0.0001) of CD29 and CD44 expression in hBM-MSCs cultured on MHA/Coll scaffold in comparison to ctrl-MSC was observed under both G and MG conditions, at 7, 14, and 21 days (Figure 4A). On the contrary, the reduction (*p* < 0.001) of CD29 and CD44 expression observed in induced-MSC under G condition was abolished in simulated MG at every time point of exposure. As expected, ctrl-MSC did not show any significant change in the expression of the selected markers under both G and MG conditions. The obtained results confirm the ability of MHA/Coll to induce hBM-MSCs differentiation even under extreme conditions of MG up to 21 days, therefore suggesting its potential to counteract bone loss induced by long-duration MG exposure.

The observed changes in the expression of hBM-MSCs surface markers were further explored by flow cytometry. hBM-MSc are negative for CD45 and positive for CD29 cell surface markers, these criteria were used to analyze hBM-MSCs differentiation under G or MG conditions after 21 days (Figure 4B), a decreasing in CD29 is related to MSCs differentiation status. Figure 4B shows CD45-/CD29+ hBM-MSCs under both G and MG conditions. In details, cells cultured on MHA/Coll scaffold showed a higher level of differentiation status both after G and MG conditions (red; plot 5 and 6, respectively) in comparison to induced-MSC under G condition (gray, plot 3). Moreover, the plot analysis of induced-MSC under MG condition (gray; plot 4) with high expression of CD29 suggested that cells lost their differentiation potential. As expected, ctrl-MSC did not show any significant change in the differentiation status under both G and MG conditions (black; plot 1 and 2, respectively) as positive CD29 cells. Furthermore, to evaluate the osteogenic potential of MHA/Coll under MG condition, we analyzed the expression of CD90 (thymocyte differentiation antigen-1, Thy-1), a well-known cell differentiation marker that decreases during cell differentiation toward osteogenic lineage (Wiesmann et al., 2006). To this end, CD90 expression was quantified by flow cytometry in cells cultured on MHA/Coll scaffold, induced-MSC and ctrl-MSC at 7, 14, and 21 days, under G or MG conditions. As shown in Figure 4C, the expression of CD90 was significantly reduced (*p* < 0.0001) in hBM-MSCs cultured on MHA/Coll scaffold in comparison to ctrl-MSC under both G and MG conditions, even after 21 days of treatment. On the contrary, induced-MSCs showed a significant decrease of CD90 expression (*p* < 0.001) only under G condition, the decrease did not appear in simulated MG at any time of exposure, confirming the osteogenic differentiation dysregulation due to MG exposure. As expected, ctrl-MSC did not show any significant change in the expression of the selected markers under both G and MG conditions. Intriguingly, under G condition but also under MG, CD90 suppression in cells cultured on MHA/Coll scaffold was more significant (*p* < 0.0001) compared to induced-MSC (*p* < 0.001) already after 7 days of incubation, therefore giving evidence for the enhanced osteogenic potential conferred by our scaffolds.

The observed findings demonstrate that the structure of MHA/Coll scaffold, characterized by nanostructured niche, may not only improve the differentiation into osteogenic cells under G condition, but also restore the osteogenic differentiation dysregulation induced by long-term exposure to the extreme condition of MG.

### Osteogenic Induction on Bio-Scaffolds Under Space Flight Conditions

To test the efficacy of the bioscaffolds under the extreme condition of long-term MG for osteogenic differentiation, hBM-MSCs seeded on MHA/Coll scaffold, induced-MSC and ctrl-MSC were cultured for 21 days, and the expression of 84 osteogenesis-associated genes was evaluated.

**FIGURE 5:**
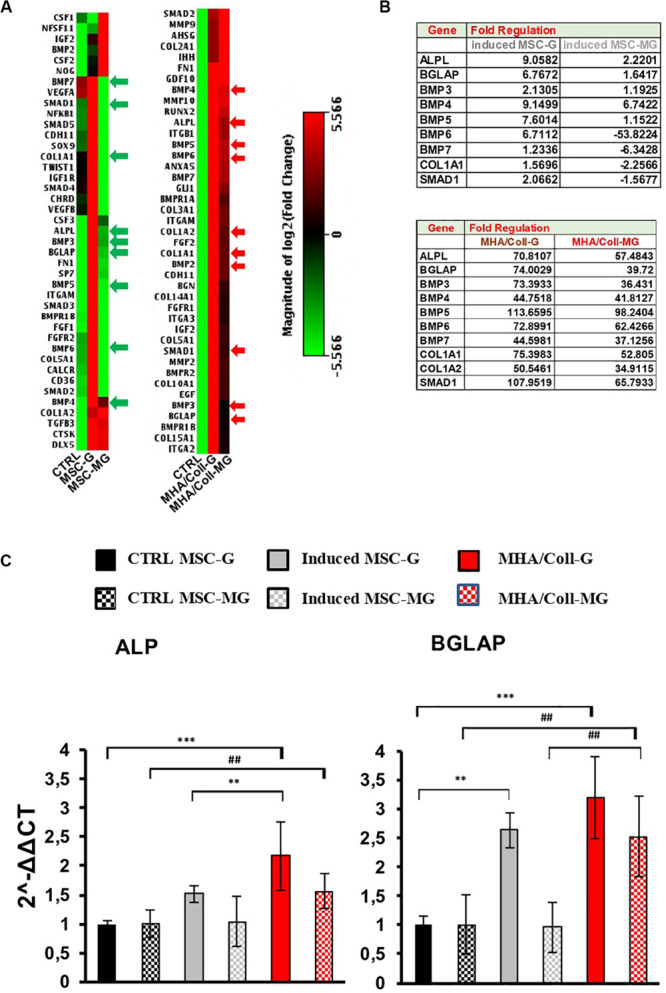
Osteo-differentiation related gene expression array in G and MG conditions. **(A)** Heat map of 84 osteo-related genes under MG conditions indicating the difference between induced-MSC and 3D MHA/Coll scaffold, showing standardized expression levels of modulated genes for the control, induced-MSC under G and induced-MSC under MG samples, respectively (red: high gene expression; green: low gene expression; black: unmodulated genes). **(B)** Tables showing the main modulate genes in comparison to the uninduced control in induced-MSC and MHA/Coll under G and MG conditions, respectively. **(C)** Osteogenic differentiation on MHA/Coll after MG. The main genes involved in osteoblast differentiation were analyzed using Real-Time PCR: alkaline phosphatase (ALP) and osteocalcin (BGLAP). Statistical differences:; **p < 0.01; ***p < 0.001, for G group vs. ctrl G; ^##^*p* < 0.01, for MG group vs. ctrl MG (Two-way ANOVA). Bars indicate the compared samples under different conditions.

The osteogenic genes differentially expressed between controls and induced cells, for both 3D (MHA/Coll) and 2D (induced-MSC) cultures, were clustered and displayed as heat map. Individual elements of the plot are colored under their relative expression values, where up- and down-regulated genes are shown as red and green squares, respectively (Figure 5A). As shown in the heat map, under G condition, cells cultured on MHA/Coll scaffold and induced-MSC exhibit an evident up-regulation of osteo-differentiation genes compared to control-MSC with different potency, MHA/Coll scaffold being significantly more effective than 2D culture.

In particular, cell cultured on MHA/Coll under G condition were able to up-regulate important oste-differentiation-related genes, such as ALP, BGLAP, BMPs family, Col1a1, Col1a2, and Smad1 (*FC* > 4) (Figure 5B). Consistent with our previous results, this ability was not reverted under MG condition. On the contrary, as expected, MG down-regulated some of these osteogenic genes in induced-MSC underlining the impairment of the osteogenic differentiation induced by MG (Figure 5B).

The intrinsic MHA/Coll osteogenic properties under MG was evidenced by the over-expression of bone morphogenetic proteins (BMPs), a group of growth factors associated with the development of bone mineralization (Sykaras and Opperman, 2003). Interestingly, the osteoinductive potential of MHA/Coll was supported by the found up-regulation of Smad1 (Mothers Against DPP Homolog 1), able to mediate the signals of the BMPs family (Massague et al., 2005). Moreover, also several collagens genes were up-regulated by MHA/Coll under MG condition confirming its osteo-differentiation potential. The collagen genes which coding proteins are associated with the extracellular matrix (ECM) such as Coll1a1 and Coll1a2 are the main proteins present in the ECM of the bone.

Furthermore, to deeper investigate the impact of long-term MG exposure on osteogenic differentiation, hBM-MSCs seeded on MHA/Coll scaffold, induced-MSC and ctrl-MSC were cultured for 21 days and the expression of ALP and BGLAP was evaluated under G and MG conditions (Figure 5C). ALP and BGLAP, two main osteogenesis-associated genes, are growth factors associated with the development of bone mineralization (Roach, 1994; Orimo, 2010).

The gene expression analysis on cells cultured on MHA/Coll under MG condition, showed an increase in the gene expression of ALP (*p* < 0.01) and BGLAP (*p* < 0.01) compared to ctrl-MSC. Moreover, the significant difference in the gene expression of BGLAP between cell cultured on MHA/Coll and induced-MSC under MG conditions (*p* < 0.01) confirmed the superior ability of MHA/Coll scaffold in inducing the osteo-differentiation of hBM-MSCs, being significantly more potent than induced-MSC (2.5-fold).

On the contrary, induced-MSC under MG condition showed the lower expression gene levels under MG condition, underlining the dysregulation effect played by MG. These findings are consistent with a large body of literature describing the dysregulation of osteoblast and osteoclasts induced by MG exposure, with a consequent decrease of bone growth and mineralization (Carmeliet et al., 1997; Bucaro et al., 2004; Blaber E.A. et al., 2014).

The osteogenic gene expression profiling played in MG suggested that MHA/Coll can influence hBM-MSCs at the molecular level also in MG. Together with our current findings, this suggests that MHA/Coll is able to transfer key cues at the nanoscale to hBM-MSCs, which overall results in an increased osteodifferentiation in MG conditions. Unlike MHA/Coll, we found that MG is able to cause a dysregulation of induced-MSCs with a downregulation of osteogenic markers and genes. The observed findings, confirm that MHA/Coll scaffolds are able to compensate the dysregulation of the osteogenesis caused by MG conditions, therefore maintaining an excellent osteogenic and osteoconductivity properties under the long-term MG extreme conditions.

## Conclusion

In conclusion, considering that during long-term space flights astronauts experience MG conditions causing bone loss and a higher risk of bone fractures, we here applied MG as a model to challenge the effectiveness of new scaffolds designed for bone regeneration in counteracting bone loss.

To this aim, encouraged by our study on the bioinspired 3D MHA/Coll scaffold ability to promote bone mass formation, we here evaluated the osteoinductive potential of this nanostructure in the extreme physiological condition of long-term MG. To this end, we present an integrative study investigating the osteogenic differentiation of hBM-MSCs cultured on the bio-inspired 3D scaffold MHA/Coll under long-term MG simulation using a RPM. The obtained findings reveal that the peculiar scaffold nanostructured niche cannot only improve the differentiation into osteogenic cells under static condition, but also restore the osteogenic differentiation dysregulation induced by long-term exposure to MG, despite the negative remodeling effect played by the latter on the scaffold.

Overall, the presented results demonstrate the ability of MHA/Coll to counteract bone dysfunction after prolonged space flights and weightlessness, confirming its eligibility as a material of choice to promote new bone formation. Despite a wide variety of bone-nano-scaffolds have been proposed, the investigation of the effect of microgravity on their osteoinduction capacity is still in its infancy (Koç et al., 2008; He et al., 2015; Koç Demir et al., 2018). Moreover, a significant difference with our study is that the experiments were carried out using rat bone marrow mesenchymal stem cells or human dental pulp stem cells, while we used human bone marrow-derived mesenchymal stem cells. It must be also noted that such experiments were not carried out using a more sophisticated system such as RPM as in our study, but a single-axis rotary cell culture system or bioreactors. Therefore, we here propose MG as a model to apply in future studies for the development of scaffolds suitable for bone regenerative medicine and tissue engineering, opening new challenges among nano-scientists for the design of bioinspired scaffolds in bone regeneration.

## Data Availability Statement

The datasets generated for this study are available on request to the corresponding author.

## Author Contributions

EA, LF, and SM performed and analyzed the experiments. EA, LF, and LD wrote the manuscript with contribution from all the authors. EA, BZ, AY, MR, PP, and ET analyzed the data and performed the study. LD conceived the idea. All authors contributed to the article and approved the submitted version.

## Conflict of Interest

The authors declare that the research was conducted in the absence of any commercial or financial relationships that could be construed as a potential conflict of interest.

## Footnotes

1 http://pcrdataanalysis.sabiosciences.com/pcr/arrayanalysis.php
