# Supplementary material for: Bioinspired Scaffold Action Under the Extreme Physiological Conditions of Simulated Space Flights: Osteogenesis Enhancing Under Microgravity
Source: Front Bioeng Biotechnol. 2020 Jul 8;8:722. doi: 10.3389/fbioe.2020.00722 (PMC7362936; doi:10.3389/fbioe.2020.00722)
Supplement: Supplementary file 1 [file Image_1.pdf]

Supplementary Material

1 Supplementary Figures

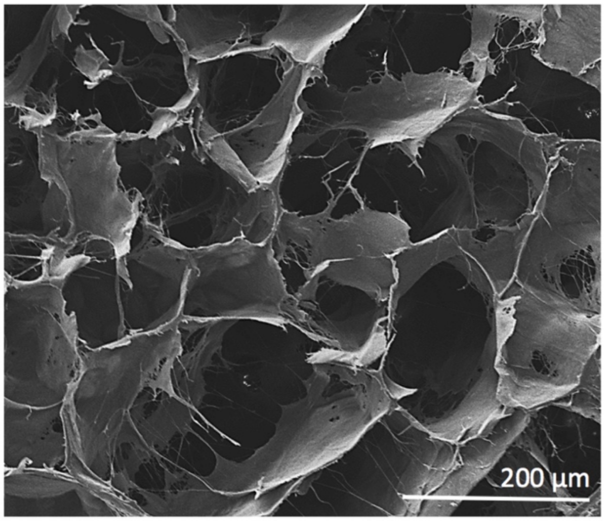

Supplementary Figure 1. Scaffold structure characterization by SEM of Coll.
